# Common Variants in the *COL4A4* Gene Confer Susceptibility to Lattice Degeneration of the Retina

**DOI:** 10.1371/journal.pone.0039300

**Published:** 2012-06-19

**Authors:** Akira Meguro, Hidenao Ideta, Masao Ota, Norihiko Ito, Ryuichi Ideta, Junichi Yonemoto, Masaki Takeuchi, Riyo Uemoto, Tadayuki Nishide, Yasuhito Iijima, Tatsukata Kawagoe, Eiichi Okada, Tomoko Shiota, Yuta Hagihara, Akira Oka, Hidetoshi Inoko, Nobuhisa Mizuki

**Affiliations:** 1 Department of Ophthalmology and Visual Science, Yokohama City University Graduate School of Medicine, Yokohama, Kanagawa, Japan; 2 Ideta Eye Hospital, Kumamoto, Japan; 3 Department of Legal Medicine, Shinshu University School of Medicine, Matsumoto, Nagano, Japan; 4 Okada Eye Clinic, Yokohama, Kanagawa, Japan; 5 Division of Molecular Life Science, Department of Genetic Information, Tokai University School of Medicine, Isehara, Kanagawa, Japan; The Children’s Hospital of Philadelphia, United States of Americs

## Abstract

Lattice degeneration of the retina is a vitreoretinal disorder characterized by a visible fundus lesion predisposing the patient to retinal tears and detachment. The etiology of this degeneration is still uncertain, but it is likely that both genetic and environmental factors play important roles in its development. To identify genetic susceptibility regions for lattice degeneration of the retina, we performed a genome-wide association study (GWAS) using a dense panel of 23,465 microsatellite markers covering the entire human genome. This GWAS in a Japanese cohort (294 patients with lattice degeneration and 294 controls) led to the identification of one microsatellite locus, D2S0276i, in the collagen type IV alpha 4 (*COL4A4*) gene on chromosome 2q36.3. To validate the significance of this observation, we evaluated the D2S0276i region in the GWAS cohort and in an independent Japanese cohort (280 patients and 314 controls) using D2S0276i and 47 single nucleotide polymorphisms covering the region. The strong associations were observed in D2S0276i and rs7558081 in the *COL4A4* gene (*P*c = 5.8×10^−6^, OR = 0.63 and *P*c = 1.0×10^−5^, OR = 0.69 in a total of 574 patients and 608 controls, respectively). Our findings suggest that variants in the *COL4A4* gene may contribute to the development of lattice degeneration of the retina.

## Introduction

Lattice degeneration of the retina is a vitreoretinal disorder characterized by a visible fundus lesion that predisposes the patient to retinal tears and detachment. The prevalence of lattice degeneration of the retina in the fellow eye of patients with retinal detachments is estimated to be 35% [1]. Lattice degeneration is characterized by sharply demarcated oval or round areas that are oriented circumferentially and are associated with liquefaction of the overlying vitreous gel and with firm vitreoretinal adhesions along the edges of the lesions. Vitreous traction on these areas after posterior vitreous detachment is often responsible for retinal tears. Lesions clinically and histopathologically indistinguishable from isolated lattice degeneration of the retina have been observed in various hereditary disorders associated with retinal detachment.

The prevalence of lattice degeneration ranges from 6% to nearly 11% in the general populations and reaches a maximum during the second decade of life [2]–[4]. There is no statistically significant difference in prevalence between men and women, and left and right eyes are involved with equal frequency [2], [3]. The prevalence of lattice degeneration of the retina is not related to race, and it has been reported to be common in Caucasians [2], Asians [5], [6], and Africans [7]. Although the etiology of familial retinal detachment associated with lattice degeneration is thought to be polygenic and/or multifactorial [8], the pathophysiological mechanism and underlying genetic factors remain unknown.

In this study, we performed a genome-wide association study (GWAS) using 23,465 microsatellite (MS) markers covering the entire human genome to detect regions associated with susceptibility to lattice degeneration of the retina. This MS set has exhibited great detection power in case-control association studies [9]–[14].

## Results

### Genome-wide MS Genotyping

**Figure 1:**
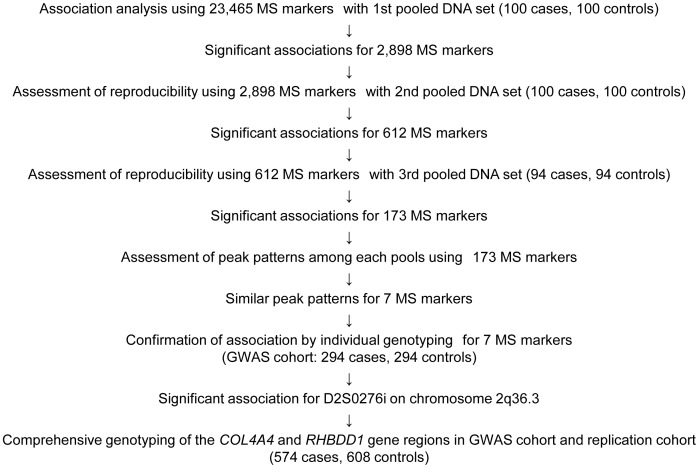
Flowchart of this genome-wide association study (GWAS) of lattice degeneration of the retina, with 23,465 microsatellite (MS) markers.

In order to reduce the type I error rate of this study, we carried out three rounds of pooled DNA screening using three independent case-control populations containing the same number of patients with essential lattice degeneration and normotensive healthy individuals (stage 1, 100; stage 2, 100; stage 3, 94). In the first screen, 2,898 markers had significantly different frequencies between cases and controls; these 2,898 markers were further analyzed in the second and third screenings using different sets of cases and controls. After the second and third screenings, 173 markers remained positive with seven markers ascribed to six distinct loci exhibiting similar peak patterns in the first, second, and third case and control pools (Figure 1).

To confirm the associations detected for these seven markers, we individually genotyped each marker in the same set of 588 screened individuals. Six of these seven markers showed statistically significant differences (*P*<0.05) after this individual screening step; however, only one marker, D2S0276i, remained statistically significant after correcting for multiple comparisons and displayed a strong association with lattice degeneration of the retina (*P*<2.1×10^−6^, corrected *P* (*P*c) <1.9×10^−5^; Table S1). We validated this association in an independent Japanese cohort of 280 patients and 314 controls. Combined analysis in the two cohorts revealed the strongest association (*P*<6.5×10^−7^, *P*c <5.8×10^−6^), and the allelic frequency of the 330-bp amplicon of D2S0276i with 12 AAT repeats ([AAT]_12_) was decreased in patients compared to controls (odds ratio (OR)  = 0.63; Table 1).

**Table 1:** Allelic association results for D2S0276i and nine SNPs in the *COL4A4* and *RHBDD1* gene regions.

					GWAS stage			Replication stage			Combined stage				
					Minor allele frequency, %			Minor allele frequency, %			Minor allele frequency, %				
	Position			Alleles	Cases	Controls		Cases	Controls		Cases	Controls			
SNP	(Build 37.1)	Gene	Location	(1>2)	(n = 294)	(n = 294)	*P*	(n = 280)	(n = 314)	*P*	(n = 574)	(n = 608)	*P*	*P*c	OR
rs4675115	227,769,123	*RHBDD1*	Intron	G>C	45.2	52.7	0.010	51.4	48.4	0.30	48.3	50.5	0.28	1.00	0.91
rs4675124	227,802,354	*RHBDD1*	Intron	A>G	35.5	45.7	3.7E-04	42.9	41.7	0.68	39.1	43.6	0.027	0.42	0.83
rs4129886	227,821,020	*RHBDD1*	Intron	T>A	20.7	14.3	0.0039	17.5	17.4	0.97	19.2	15.9	0.044	0.57	1.25
D2S0276i	227,893,485	*COL4A4*	Intron	[AAT]_12_	21.3	33.6	2.1E-06	25.4	31.4	0.022	23.3	32.5	6.5E-07	5.8E-06	0.63
rs3769641	227,922,321	*COL4A4*	Intron	A>G	21.2	15.3	0.0094	18.0	17.6	0.84	19.7	16.5	0.046	0.60	0.81
rs3923084	227,948,726	*COL4A4*	Intron	A>T	22.9	17.8	0.028	21.4	19.0	0.31	22.2	18.4	0.023	0.37	0.79
rs2229814	227,954,599	*COL4A4*	Mis-sense	G>A	54.8	46.6	0.0049	52.7	46.8	0.043	53.8	46.7	6.0E-04	0.015	1.33
rs4389330	227,957,483	*COL4A4*	Intron	G>C	52.4	42.8	0.0010	51.8	44.2	0.0091	52.1	43.5	3.1E-05	4.0E-04	1.41
rs7558081	227,958,469	*COL4A4*	Intron	T>C	43.2	51.9	0.0029	44.3	53.8	0.0010	43.7	52.9	6.9E-06	1.0E-05	0.69
rs6718820	228,038,804	*COL4A4*	Intron	C>A	25.7	30.9	0.0498	30.7	32.3	0.56	28.1	31.6	0.068	0.72	0.85

1, major allele; 2, minor allele; OR, odds ratio; SNP, single nucleotide polymorphism.

Position is distance from short arm telomere. P values were calculated by χ^2^ test 2×2 contingency table. We corrected *P* values (*P*c) of D2S0276i and 5 SNPs in the combined stage for multiple testing by Bonferroni's method and Haploview program using 10,000 permutations, respectively.

### Single Nucleotide Polymorphism (SNP) Genotyping of the D2S0276i Region

**Figure 2:**
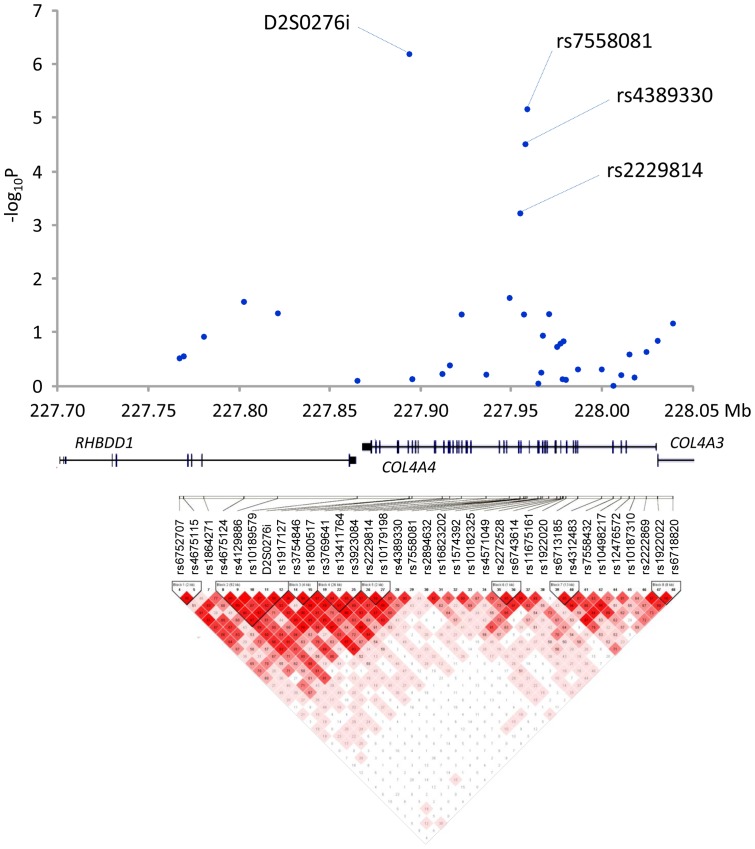
Association analysis of single nucleotide polymorphisms (SNPs) across the *COL4A4* and *RHBDD1* gene regions in 574 patients and 608 controls. The upper panels depict distribution of association results of D2S0276i and SNPs in *COL4A4* and *RHBDD1*. The results of monomorphic SNPs are not shown. The lower panels show the linkage disequilibrium structure in the *COL4A4* and *RHBDD1* regions; brighter red indicates higher D’.

D2S0276i is located in the collagen type IV alpha 4 (*COL4A4*) gene on chromosome 2q36.3 and its linkage disequilibrium. (LD) appears to extend to the next gene, rhomboid domain containing 1 (*RHBDD1*). To dissect this region further, we selected a collection of evenly spaced SNPs (coding and non-coding) covering 350 kb around *COL4A4* and *RHBDD1*. We genotyped samples from 1182 individuals (588 for the GWAS and 594 for replication) using 47 SNPs (Table 1, Table S2, Figure 2). Of these SNPs, 34 were genotyped successfully, whereas 12 missense and 1 nonsense SNPs turned out to be monomorphic. Among these 34 SNPs, nine (rs4675115, rs4675124, rs4129886, rs3769641, rs3923084, rs2229814, rs4389330, rs7558081, and rs6718820) exhibited an association with lattice degeneration of the retina at *P*<0.05 in the GWAS cohort. rs2229814, rs4389330 and rs7558081 showed evidence of replication in the independent cohort. The strongest association was observed at rs7558081 in the combined cohort (*P*<6.9×10^−5^, *P*c<1.0×10^−5^), and minor allele C of rs7558081 was decreased in patients compared to controls (OR = 0.69).

### Linkage Disequilibrium and Haplotype Analysis

Figure 2 shows the overall LD patterns for D2S0276i and the 34 SNPs genotyped successfully in 1182 individuals. Strong LD was observed throughout the ∼200-kb region including D2S0276i and rs7558081 (from rs6752707 to rs7558081), and we therefore identified this region as a candidate region for susceptibility to lattice degeneration of the retina. D2S0276i exhibited a strong LD with rs4389330 (*D*’ = 0.80), rs2229814 (*D*’ = 0.79) whereas LD between D6S0276i and rs7558081 was a moderate level (*D*’ = 0.44). rs7558081, rs4389330 and rs2229814 were in high LD (*D*’>0.80).

To elucidate whether association of rs7558081, rs4389330 and rs2229814 with the disease was due to LD with D2S0276i, we stratified the patient and control populations according to the presence of allele [AAT]_12_ of D2S0276i (Table 2). In 343 patients and 301 controls lacking [AAT]_12_, only rs7558081 showed a weak association with the disease (*P* = 0.021, OR = 0.77) whereas rs4389330 and rs2229814 did not display significant associations with the disease. Eleven haplotypes consisting of D2S0276i, rs2229814, rs4389330 and rs7558081 were observed, and the frequencies of the haplotypes including [AAT]_12_ of D2S0276i decreased in patients independent of the status of the rs2229814, rs4389330 and rs7558081 alleles (Table 3).

**Table 2:** Allele frequencies of rs2229814, rs4389330 and rs7558081 in carriers and non-carriers of [AAT]_12_ allele of D2S0276i.

		[AAT]_12_ non-carriers, allele frequency, %			
SNP	Allele	Cases(n = 343)	Controls(n = 301)	*P*	OR
rs2229814	A	65.8	63.6	0.40	1.10
rs4389330	C	63.6	59.1	0.094	1.21
rs7558081	C	37.3	43.8	0.021	0.77

OR, odds ratio.

*P* values were calculated by χ^2^ test 2×2 contingency table.

**Table 3:** Haplotype frequencies of D2S0276i, rs2229814, rs4389330 and rs7558081 of the *COL4A4* gene.

	Haplotype frequency, %			
Haplotype	Cases(n = 574)	Controls(n = 608)	*P*	OR
(D20276i, rs2229814, rs4389330 and rs7558081)				
[AAT]_10_ : A : C : T	0.244	0.187	0.0011	1.39
[AAT]_12_ : G : G : C	0.164	0.231	2.6E-05	0.64
[AAT]_14_ : A : C : T	0.180	0.139	0.010	1.34
[AAT]_10_ : G : G : C	0.127	0.154	0.047	0.79
[AAT]_13_ : G : G : C	0.095	0.061	0.0024	1.60
[AAT]_12_ : G : G : T	0.047	0.056	0.30	0.82
[AAT]_13_ : A : C : T	0.048	0.039	0.32	1.23
[AAT]_12_ : A : C : T	0.019	0.029	0.11	0.65
[AAT]_10_ : A : G : C	0.014	0.029	0.012	0.47
[AAT]_14_ : G : G : C	0.014	0.015	0.77	0.90
[AAT]_13_ : A : C : C	0.009	0.011	0.50	0.76

OR, odds ratio.

Haplotypes with frequency less than 1% are not listed. *P* values were calculated by χ^2^ test 2×2 contingency table.

### Expression Analysis of COL4A4

D2S0276i is located in intron 41 of *COL4A4*. Since intronic polymorphisms may significantly affect *COL4A4* expression, we investigated the association between the presence of D2S0276i [AAT]_12_ and the level of *COL4A4* expression using real-time quantitative RT-PCR. The expression of COL4A4 mRNA changed dependent on the presence of [AAT]_12_, but not significantly, suggesting that [AAT]_12_ may affect expression levels (Figure S1).

## Discussion

Here we performed a GWAS of lattice degeneration of the retina by multistep screening with 23,465 well-characterized MS markers to cover the euchromatic area (∼90%) of the human genome at average intervals of 115.1 kb; this investigation identified *COL4A4* as a novel candidate gene for lattice degeneration of the retina. *COL4A4* encodes the alpha 4 (IV) chain of type IV collagen, a flexible protein. This chain combines with the alpha 3 and alpha 5 chains to make a complete type IV collagen molecule. Type IV collagen molecules attach to each other to form complex protein networks that make up a large portion of the basement membranes, thin sheet-like structures that separate and support cells in many tissues.

Pathogenic features of lattice degeneration of retina include liquefaction of the adjacent vitreous humor, absence of vitreoretinal attachments, absence of the internal limiting membrane over the lesions, and vitreous condensation with a firm vitreoretinal attachment at the lesion margins. Pathogenesis may be due to a developmental abnormality involving the internal limiting membrane of the retina [15], which consists of the basal lamina of Müller’s cells and their basement membrane. Type IV collagen alpha 3–5 networks play an especially important role in the basement membranes of the kidney, inner ear, and eye [16]; *COL4A4* mutations have been found to cause Alport syndrome and thin basement membrane nephropathy [17]–[22]. Although the pathogenesis of lattice degeneration of the retina remains poorly understood, the basement membrane makes up a major retinal structure. Vitreoretinal degeneration is complicated by retinal detachment in Alport syndrome [23], leading us to speculate that aberrations in *COL4A4* may be involved in retinal thinning in cases of lattice degeneration of the retina.

The strongest associations with the disease that we detected occurred at D2S0276i in intron 41 and rs7558081 intron 20 of *COL4A4*, and secondary signals were observed at rs4389330 (intron 20) and rs2229814 (exon 21), with the stratification and haplotype analyses suggesting that the association with rs7558081, rs4389330 and rs2229814 was due to LD with D2S0276i and was not a primary association. Therefore, D2S0276i or its neighboring polymorphism may be a causative factor for lattice degeneration of the retina. To clarify the primary association, it will be necessary to further explore the LD region of D2S0276i by a re-sequencing analysis with D2S0276i [AAT]_12_-positive patients. Since intronic polymorphisms can significantly affect gene expression, we surmised that the disease-associated allele of D2S0276i [AAT]_12_ may regulate *COL4A4* expression. However, we detected no distinct association between [AAT]_12_ and gene expression in this study although the expression levels observed in whole blood may not reflect the levels in eye tissues.

This study is the first to comprehensively investigate susceptibility genes for lattice degeneration of the retina. *COL4A4* on chromosome 2q36.3 was strongly associated with disease susceptibility in the Japanese population, with allele [AAT]_12_ of D2S0276i exhibiting the strongest association. To confirm our findings, future validation studies with other ethnic populations are needed and if the validation succeeds, [AAT]_12_ may serve as a useful genetic marker for the diagnosis of lattice degeneration of the retina. In addition, we could not demonstrate any functional roles of *COL4A4* polymorphisms in disease development. Thus, functional studies are also required to clarify how *COL4A4* polymorphisms contribute to the pathogenesis of lattice degeneration of the retina.

## Materials and Methods

### Participants

A total of 574 unrelated patients with lattice degeneration of the retina and 608 unrelated healthy controls, all of Japanese descent, were enrolled in this study. The subjects were recruited from Yokohama City University, Ideta Eye Hospital, Yonemoto Eye Clinic, Nanbu Hospital, and Tokai University. Blood samples were collected from the patients who were scheduled for surgery for retinal detachment caused by lattice degeneration. Diagnosis of lattice degeneration was made with indirect ophthalmoscopy and scleral indentation by retina specialists via one or a combination of the following inclusion criteria: the observation of lattice-like white line changes in the crossing retinal vessels; the presence of snail-track variations; alteration in pigmentation; the presence of ovoid or linear reddish craters; localized round, oval, or linear retinal thinning; attachment of condensed vitreous fibers to the edges of the lesion. In spite of variations in pigmentation or other morphological features, the lesion was regarded as lattice degeneration when the examiner encountered an abrupt, discrete irregularity of the otherwise smooth retinal surface at the borders of the lesion. All control participants were healthy volunteers unrelated to each other or to the patients. This study methodology complied with the guidelines of the Declaration of Helsinki and was approved by the ethics committees of Yokohama City University, Ideta Eye Hospital, Yonemoto Eye Clinic, Nanbu Hospital, and Tokai University. We obtained informed written consent from all participants who donated DNA samples for this analysis. Personal identities associated with medical information and blood samples were eliminated and replaced with anonymous identities at each recruiting institution.

### Pooled DNA Construction

DNA pooling was performed as previously [24] with a slight modification [25]. Genomic DNA was extracted from peripheral blood lymphocytes using the QIAamp DNA Blood Maxi Kit (QIAGEN) under standardized conditions. Following extraction, DNA degradation and RNA contamination were assessed via 0.8% agarose gel electrophoresis; DNA was quantified using a double-standard DNA quantification kit (PicoGreen, Molecular Probes) as previously described [9], [26]. To minimize pipetting error, all DNA samples were measured side by side by the same technician using one set of calibrated pipettes. The concentration of the genomic DNA samples was adjusted to 8 ng/µl and the samples were divided equally into three case pools and three control pools, with each pool containing equal numbers of men and women. The pooled DNA template for MS typing was prepared immediately by mixing equal numbers of patient and control DNAs (100 at the first stage, 100 at the second stage, and 94 at the third stage). Multiple peak patterns from the pooled DNA sample mirrored the distribution of allele frequencies in the subject samples [24]. To confirm the suitability of pool DNA as a time- and cost-saving alternative to individual DNA typing, we compared the allelic distribution of three MS markers typed using pooled vs. individual DNA (n = 100). Allelic distributions were assessed using Fisher’s exact test. No significant differences in allele frequencies were found between pooled and individual DNAs, thus validating this technique for large-scale use.

### Genome-wide MS Genotyping

All MS markers and methods for MS genotyping used in this study have been previously described [9]. PCR primers were designed with a homogenous annealing temperature of 57°C. Forward primers were labeled at the 5′ end with the fluorescent reagents 6-FAM or HEX (Applied Biosystems). PCRs of pooled DNAs were performed in 20-µl reactions containing 24 ng of pooled DNA, 0.5 U AmpliTaq Gold DNA polymerase (Applied Biosystems), 2 µl 10× reaction buffer (100 mM Tris-HCl [pH 8.3], 500 mM KCl, 15 mM MgCl_2_), 2 µl dNTPs (2.5 mM each), and 20 pmol of forward and reverse primers. The amplification conditions consisted of initial denaturation at 96°C for 9 min, annealing at 57°C for 1 min, and extension at 72°C for 1 min, followed by 30 cycles of denaturation at 96°C for 45 sec, annealing at 57°C for 45 sec, and extension at 72°C for 1 min in a GeneAmp PCR system 9700 thermal cycler (Applied Biosystems). PCR of individual DNA was carried out in 20-µl reactions containing 1 ng of genomic DNA, 0.5 U AmpliTaq Gold DNA polymerase, 2 µl 10× reaction buffer, 2 µl dNTPs (2.5 mM each), and 20 pmol of each primer. The amplification conditions were essentially the same as described above except that 40 cycles were used and a final extension of 5 min at 72°C was carried out after 40 cycles. The amplified products were denatured in formamide (Hi-Di, Applied Biosystems) at 95°C for 3 min and separated on a 3700 DNA analyzer (Applied Biosystems). Various data about the markers, such as amplified peak positions and heights, were manually extracted by the PickPeak and MultiPeaks programs developed by Applied Biosystems Japan.

In the first screen with 100 cases and 100 controls, we employed a total of 23,465 MS markers. Markers demonstrating a significant association (*P*<0.05) with lattice degeneration of the retina were subjected to a second screen of another set of 100 cases and 100 controls. Markers exhibiting a significant association in the second screen were subjected to a third screen with another distinct 94 cases and 94 controls. The markers that tested positive in all three screens were assessed for similar peak patterns in the first, second, and third case and control pools; the identified markers were subjected to the individual DNA screen and were genotyped in 588 individuals (the same 294 cases and 294 controls as mentioned above). After the individual DNA screen, we tested the MS D2S0276i, which showed a strong association with lattice degeneration of the retina, in an independent Japanese cohort of 280 patients and 314 controls (Figure 1).

### SNP Genotyping of the COL4A4 and RHBDD1 Gene Regions

SNPs in the *COL4A4* and *RHBDD1* gene regions were selected from HapMap Japanese data (MAF ≥20%, pairwise *r* ^2^≥0.7, P_HWE_ ≥0.05). In addition, known non-synonymous SNPs in the *COL4A4* and *RHBDD1* gene regions were also selected. Forty-seven SNPs were selected and used to detect significant differences between case and control samples (Table S2).

In this SNP genotyping to validate the MS-based GWAS results, the GWAS cohort (the same 294 cases and 294 controls) and an independent Japanese cohort (280 patients and 314 controls) were used. SNP genotyping was performed using TaqMan® SNP Genotyping Assays according to the manufacturer’s instructions. Reactions were carried out with the ABI GeneAmp® System 9700 thermal cycler and the ABI PRISM® 7900HT Sequence Detection System (Applied Biosystems) using a 384-well block module for measuring fluorescence. SDS software version 2.0 was used for allelic discrimination (Applied Biosystems). Ten nanograms of genomic DNA were used as template for PCR amplification.

### Real-time Quantitative RT-PCR

Whole blood (2.5 mL) was collected in PAXgene Blood RNA tubes (Becton Dickinson) and gently mixed; tubes were incubated at room temperature for 2 hours, and total RNA was extracted using the PAXgene Blood RNA Kit (QIAGEN) according to the manufacturer’s protocol. cDNA was synthesized using the SuperScript™ II Reverse Transcriptase (Invitrogen). Quantitative RT-PCR of the *COL4A4* gene was performed using the StepOnePlus™ Real-Time PCR System (Applied Biosystems) with TaqMan gene expression assays (Assay ID: Hs01011885_m1, Applied Biosystems), the THUNDERBIRD™ Probe qPCR mix (Toyobo), and ROX reference dye (Toyobo). *COL4A4* expression levels were normalized to those of 18S ribosomal RNA (Assay ID: Hs99999901_s1, Applied Biosystems) for each individual.

### Statistical Analysis

For MS genotyping, statistical significance of differences was assessed by Fisher’s exact test using the 2×2 and 2×m (where m is the number of alleles) contingency tables for each allele. Allele frequencies in pooled DNA stages were estimated from the height of the peaks; each allele frequency was determined by dividing the height of each allele peak by the summed height of all allele peaks. For the exact probability test, the Markov chain/Monte Carlo simulation method was employed to execute the Fisher’s exact test for the 2×m contingency table. The smallest *P* value for each marker was selected. In the individual MS genotyping stage, the probability of association was corrected by the Bonferroni’s method, i.e. by multiplying the obtained *P* values with the number of observed alleles at the locus (corrected *P* [*Pc*] value). Association analyses for the SNP genotyping stage were carried out using Haploview 4.1 [27] and obtained *P* values were corrected for multiple testing by a permutation test (10,000 iterations) using Haploview. Haploview 4.1 was also used to compute pairwise LD statistics. Haplotype frequencies in the multi-locus analyses were calculated using PyPop 0.7.0 [28]. Haplotype frequencies were estimated using the iterative Expectation-Maximization algorithm. LD was measured using Hedrick’s multiallelic *D’* statistic [29]. Differences in *COL4A4* mRNA expression levels were analyzed via the Mann**-**Whitney U test.

## Supporting Information

Figure S1 **Expression analysis of** ***COL4A4*** **mRNA stratified by presence of D2S0276i [AAT]_12_.** Bars represent mean values and standard deviations of mRNA levels.
(PPT)

Table S1 **Seven microsatellite markers used in the individual DNA screening.** OR, odds ratio. *Each allele was named by the size of its amplification. *P* values were calculated by χ2 test 2×2 contingency table. These *P* values were corrected by multiplying by the number of observed alleles at the locus (*P*c value).
(XLS)

Table S2 **Allelic association results for D2S0276i and 47 SNPs in the** ***COL4A4*** **and** ***RHBDD1*** **gene regions.** 1, major allele; 2, minor allele; OR, odds ratio; SNP, single nucleotide polymorphism. Position is distance from short arm telomere. *P* values were calculated by χ^2^ test 2×2 contingency table. We corrected *P* values (*P*c) of D2S0276i and 47 SNPs in the combined stage for multiple testing by Bonferroni's method and Haploview program using 10,000 permutations, respectively.
(XLS)
