# Supplementary material for: Common Variants in the COL4A4 Gene Confer Susceptibility to Lattice Degeneration of the Retina
Source: PLoS One. 2012 Jun 19;7(6):e39300. doi: 10.1371/journal.pone.0039300 (PMC3378527; doi:10.1371/journal.pone.0039300)
Supplement: Figure S1 — Expression analysis of COL4A4 mRNA stratified by presence of D2S0276i [AAT]12. Bars represent mean values and standard deviations of mRNA levels. (PPT) [file pone.0039300.s001.ppt]

## Slide 1
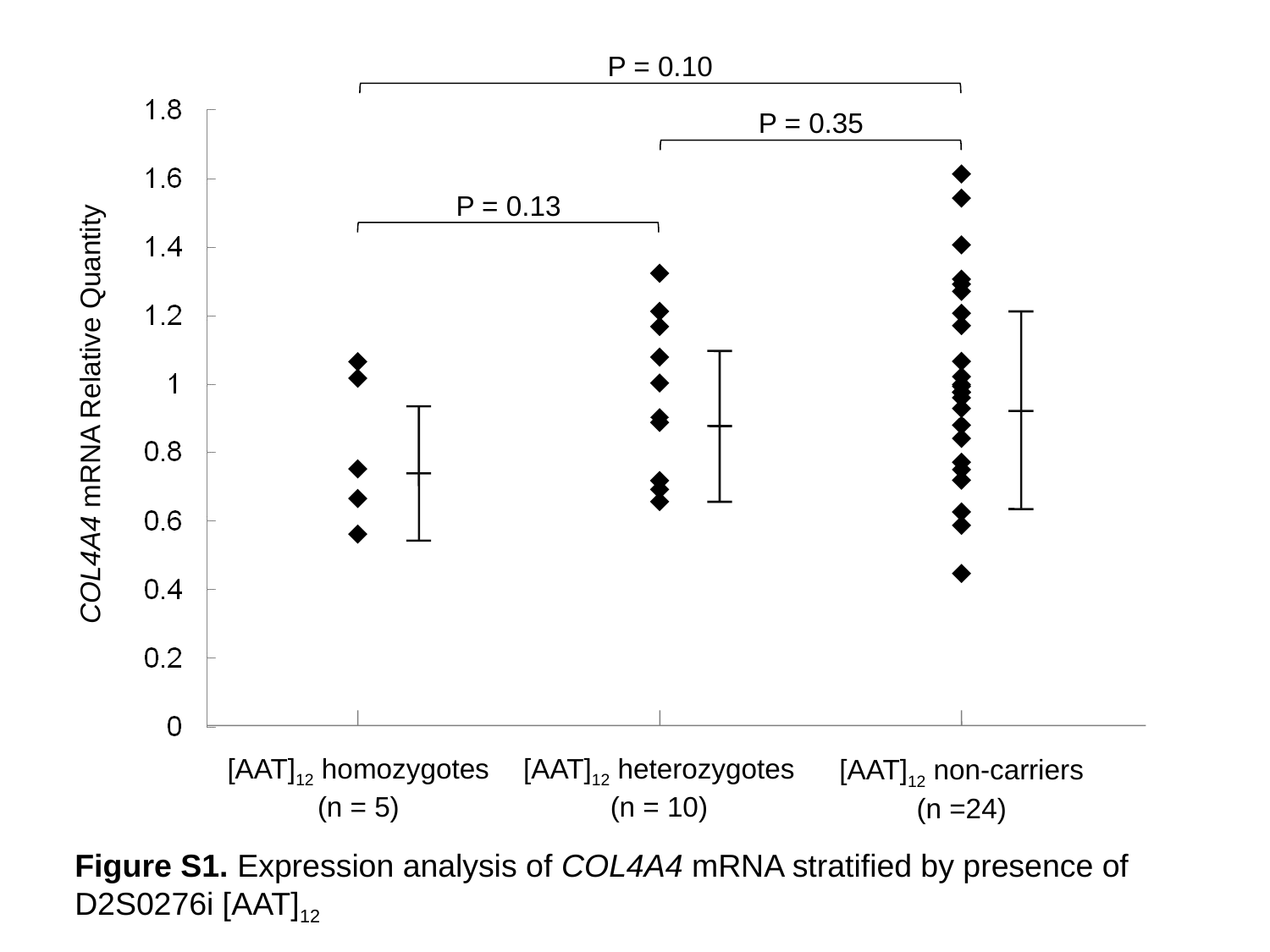

P = 0.10
P = 0.35
P = 0.13
COL4A4 mRNA Relative Quantity
[AAT]12 heterozygotes
(n = 10)
[AAT]12 homozygotes
(n = 5)
[AAT]12 non-carriers
(n =24)
Figure S1. Expression analysis of COL4A4 mRNA stratified by presence of D2S0276i [AAT]12
